# Interleukin-1 Blockers in Recurrent and Acute Pericarditis: State of the Art and Future Directions

**DOI:** 10.3390/medicina60020241

**Published:** 2024-01-30

**Authors:** Antonella Gallo, Maria Grazia Massaro, Sara Camilli, Silvino Di Francesco, Laura Gerardino, Elena Verrecchia, Ludovico Luca Sicignano, Francesco Landi, Raffaele Manna, Massimo Montalto

**Affiliations:** 1 Fondazione Policlinico Universitario “A. Gemelli” IRCCS, 00168 Rome, Italy; laura.gerardino@policlinicogemelli.it (L.G.); elena.verrecchia@policlinicogemelli.it (E.V.); ludovicoluca.sicignano@policlinicogemelli.it (L.L.S.); francesco.landi@unicatt.it (F.L.); raffaele.manna@unicatt.it (R.M.); massimo.montalto@unicatt.it (M.M.); 2 Department of Geriatrics and Orthopedics, Università Cattolica del Sacro Cuore, 00168 Rome, Italy

**Keywords:** pericarditis, anti-IL-1 drugs, autoinflammatory

## Abstract

Diseases of the pericardium encompass a spectrum of conditions, including acute and recurrent pericarditis, where inflammation plays a pivotal role in the pathogenesis and clinical manifestations. Anti-inflammatory therapy indeed forms the cornerstone of treating these conditions: NSAIDs, colchicine, and corticosteroids (as a second-line treatment) are recommended by current guidelines. However, these medications come with several contraindications and are not devoid of adverse effects. In recent years, there has been an increased focus on the role of the inflammasome and potential therapeutic targets. Recurrent pericarditis also shares numerous characteristics with other autoinflammatory diseases, in which interleukin-1 antagonists have already been employed with good efficacy and safety. The objective of this review is to summarize the available studies on the use of anti-IL-1 drugs both in acute and recurrent pericarditis.

## 1. Introduction

Pericardial diseases are a heterogeneous group of entities, ranging from acute pericarditis to asymptomatic pericardial effusion [1]. According to the latest update of the European Society of Cardiology (ESC) guidelines on pericardial diseases, published in 2015 [2], we define (a) acute pericarditis (AP) as an inflammatory pericardial syndrome, with or without pericardial effusion; (b) recurrent pericarditis (RP) as being identified by the presence of new signs and symptoms of pericardial inflammation after a first documented episode of acute pericarditis and a symptom-free interval of 4–6 weeks or longer; (c) incessant pericarditis as when pericarditis symptoms last more than 4–6 weeks, but less than 3 months, without remission; and (d) chronic pericarditis as when pericarditis symptoms manifest for more than 3 months.

The diagnosis of AP requires at least two of the following diagnostic criteria: typical chest pain; the finding of a pericardial friction rub; and new electrocardiographic findings, such as widespread ST elevation or PR depression (observed in up to 60% of cases), and the presence of pericardial effusion on echocardiogram (seen in up to 60% of cases) [2].

Precise epidemiological data for AP are lacking. The reported incidence in an urban area in Northern Italy was about of 27.7 cases per 100,000 person-years [3]. Recently, many epidemiologic studies have highlighted how SARS-CoV-2 infection increased the incidence of pericarditis at least 15 times over pre-COVID levels, although the condition remains rare [4].

Nonsteroidal anti-inflammatory drugs (NSAIDs), colchicine, and steroids (as second-line treatment) represent the mainstays of AP therapy [1,2]; however, up to 30% of patients with AP experience a recurrence, especially if not treated with colchicine, and up to 50% of recurrent patients can experience more than one recurrence [2].

To date, interleukin-1 (IL-1) antagonists (also called anti-IL-1 drugs or IL-1 blockers) are approved as a third line of therapy, only for cases of a non-infectious, steroid-dependent, and colchicine-refractory RP, with excellent results of efficacy and safety [2,5].

However, some recent evidence, although deviating from current guidelines, has shown interesting data regarding the use of these drugs as first- or second-line treatments in both recurrent and acute pericarditis, with preliminary good safety and efficacy results.

The aim of this review is to analyze the state of the art regarding the use of currently available IL-1 blockers in any line of therapy, both in AP and RP, with a special focus on their efficacy, tolerability, and safety.

## 2. Etiology and Current Treatment of Acute and Recurrent Pericarditis

AP arises from various causes, including infections (bacterial; viral, including Dengue fever; and, rarely, fungal); malignancies; and traumas—both direct injuries to the pericardium and delayed conditions, like post-myocardial infarction syndrome [2,6]. Additionally, pericardial involvement can occur in many autoimmune and autoinflammatory diseases (systemic lupus erythematosus, vasculitis, sarcoidosis, familial mediterranean fever) [7,8]. More recently, different cases of pericarditis after vaccination for COVID-19 have also been described [9,10]. However, while in developing countries the most common cause of AP is due to Mycobacterium tuberculosis, in Western European countries, the etiology of AP remains unknown in most cases, in up to 55% of cases of idiopathic AP [7,8].

These data can be partly explained by the fact that an extensive etiological investigation through pericardial fluid analysis is often not performed, and the available laboratory tests provide low sensitivity for many causes, including the viral ones. Indeed, in countries with a low prevalence of tuberculosis infection, the 2015 ESC guidelines state that an etiological investigation is not mandatory [2]. It is recommended, however, in cases exhibiting negative prognostic factors (high fever above 38 °C, subacute onset, large pericardial effusion, tamponade, etc.) or when the onset clinical presentation is highly indicative of a specific etiology [2].

The treatment of a first episode of AP consists of anti-inflammatory therapy, such as NSAIDs, which are the mainstays of AP management [2]. NSAIDs are contraindicated in case of drug allergy or intolerance; chronic renal disease (Estimated Glomerular Filtration Rate below 30 mL/min); high risk of bleeding conditions (e.g., recent gastrointestinal ulcers or concomitant anticoagulant therapy); and, in women, pregnancy beyond the 20th week of gestation [2,11,12].

Colchicine is used in combination with NSAIDs to enhance treatment responsiveness and prevent recurrences. The dosage is adjusted based on body weight (0.5 mg once if body weight is <70 kg and 0.5 mg twice daily if >70 kg) for at least three months. Other dose adjustments include patients weighting <70 Kg and those with chronic renal disease. The most common side effects from colchicine are gastrointestinal ones, mainly diarrhea [2,13,14]. Patients who do not respond or have contraindications to the first line of treatment should receive corticosteroids at low-to-moderate doses (i.e., prednisone 0.2–0.5 mg/kg/day), with a cautious step-down approach based on symptoms and C-reactive protein (CRP) levels [2]. However, several studies have indicated that the utilization of corticosteroids, whether as a first-line or second-line treatment (in patients for whom NSAIDs were ineffective), might be linked to a greater risk of recurrence compared to patients effectively treated with NSAIDs [15].

Additionally, prolonged corticosteroid use implicates the risk of several adverse effects, including non-alcoholic steatohepatitis, hyperglycemia, and osteoporosis. In order to avoid vertebral fractures, calcium, vitamin D, and bisphosphonates are recommended for patients with long-term treatment [11].

The genesis of RP is currently believed to lie halfway between autoimmune and autoinflammatory pathways [16].

On the one hand, autoimmune clues are represented by the possible occurrence of serum anti-heart (AHA), anti-nuclear (ANA), and anti-intercalated disk antibodies (AIDAs) [17], as well as the assessment of human leukocyte antigen (HLA) haplotypes predisposing to or protective against RP [18]. Conversely, RP is seen in several autoinflammatory diseases, such as familial mediterranean fever (FMF), where a dysregulation of the innate immune system is crucial to the development of the disease [19,20].

Clinically, pericarditis with an autoimmune pathway typically presents with a subacute course, a moderate increase in markers of inflammation, and a possible association with Raynaud phenomenon, arthralgias, uveitis, and sicca syndrome [15]. On the other hand, an autoinflammatory pathway usually manifests with more symptoms, higher fever, and a rise in relevant inflammatory markers [19].

The current treatment options for RP, according to the ESC guidelines, are summarized in Table 1.

## 3. The Inflammasome Complex: A Key Player in Pericardial Inflammation

Inflammasomes are essentially intracellular innate immune sensors, multiprotein complexes whose assembly and activation in the cytosol after a large variety of “danger signals”, like DAMPS (Damage-Associated Molecular Patterns) and PAMPS (Pathogen-Associated Molecular Patterns), finally leads to the synthesis of pro-inflammatory cytokines, such as interleukin-1β (IL-1β) and interleukin-18 (IL-18) [21].

The most studied inflammasome is the ubiquitous NLRP3 (NACHT, leucine-rich repeat, and pyrin domain-containing protein 3) inflammasome, also known as cryopyrin. Basically, NLRP3 is made up of a sensor (NLRP3, a member of the NOD-like receptor proteins); a scaffold protein (ASC, apoptosis-associated speck-like protein containing a COOH-terminus caspase activation and recruitment domain); and an effector, caspase-1 [22,23].

In the context of AP, an initial trigger leads to the release of DAMPs or PAMPs, which bind to cellular pattern recognition receptors (PRRs), thus activating several intracellular pathways that ultimately lead to an increase in transcription of NLRP3 inflammasome. This structure induces caspase-1 to cleave pro-IL-1β into its active form, IL-1β; the consequent systemic release of IL-1β causes recruitment of main actors of the innate immune response (neutrophils, macrophages) in the site of injury [21,24]. After cellular injury, another member of the IL-1 family, interleukin 1-alfa (IL-1α), is released by injured pericardial cells and triggers an inflammatory response by the same inflammation pathway of IL-1β [25,26,27].

In RP, some of the above-mentioned mechanisms keep on going, despite treatment with NSAIDs (typically combined with colchicine). Intriguingly, Mauro et al. have recently proved that inflammasome NLRP3 and IL-1 are inflammation culprits in RP pathogenesis, highlighting the role of NLRP3 both in human and in murine pericarditis, as well as the better response of murine-induced pericarditis to drugs antagonizing the NLRP3 pathway [28].

Overall, RP shows clear similarities with autoinflammatory diseases, a group of disorders characterized by innate immune system dysfunction [29]: in particular, FMF, Tumor necrosis factor Receptor-Associated Periodic Syndrome (TRAPS), and Adult Onset Still Disease (AOSD) [27,28,29] clinically presenting with flares with high fever, serositis, and a rise in inflammatory markers, interspersed with disease-free periods.

Of note, cytokine IL-1 has a pivotal role in sustaining autoinflammation in all the above-mentioned disorders, so that, nowadays, IL-1 blockers represent the best treatment choice for most autoinflammatory disease [29,30,31,32,33,34].

In this context, some anti-IL1 antagonists have been studied and approved also for steroid-dependent and colchicine-refractory pericarditis.

## 4. Interleukin-1 Blockers in Recurrent Pericarditis

### 4.1. Anakinra

Anakinra is a recombinant human IL-1 receptor antagonist (IL-1Ra), blocking both IL-1β and IL-1α activity [25,35]; due to its short half-life (about 2.6 h), the standard adult dose corresponds to daily administration of 100 mg subcutaneously [36].

Although only recently adopted in RP, Anakinra is approved by United States Food and Drug Administration (FDA) for the treatment of Rheumatoid Arthritis (RA), cryopyrin-associated periodic syndromes (CAPS), TRAPS, and FMF [18]. The use of Anakinra is allowed by the European Medicines Agency (EMA) for RA in combination with methotrexate when monotherapy with the latter has failed: CAPS, FMF (association with colchicine should be considered), and Still’s disease [37]. Anakinra has also been approved for patients with COVID-19 pneumonia at risk of severe respiratory failure [38]

The first double-blind, placebo-controlled, randomized trial analyzing the use of Anakinra in RP was the Anakinra-Treatment of Recurrent Pericarditis (AIRTRIP) study [39]. Twenty-one patients with a clinical story of more than three pericarditis recurrences, laboratory evidence of CRP elevation above 1 mg/dL, colchicine resistance, and corticosteroid dependence received Anakinra 2 mg/kg/day (with a maximum dose of 100 mg) for 2 months. During this phase, all patients had pain resolution and CRP reduction, leading to the discontinuation of all anti-inflammatory drugs (including steroids) by 6 weeks [39]. Patients were then randomized to Anakinra or placebo for 6 months or until recurrence. After a median follow-up of 14 months, pericarditis recurred in 9 of 10 (90%) patients assigned to placebo and only in 2 of 11 (18%) patients treated with Anakinra. Side effects of Anakinra treatment were transient and minor, mostly injection site skin reactions, occurring in 95% of patients during the first 2 months of treatment (Table 2).

Later, in 2020, the IRAP (International Registry of Anakinra for Pericarditis) study, a multicenter observational work, confirmed the efficacy and safety of Anakinra in the treatment of 224 colchicine-resistant and steroid-dependent patients affected by RP with high CRP values [40]. Patients received 100 mg daily of Anakinra for a median treatment of six months. Response to Anakinra was rapid (within one week of treatment), with clinical benefit and CRP reduction, so that steroid therapy was gradually tapered to discontinuation in most patients. Pericarditis recurrences found a 6-fold reduction, with an 11-fold reduction of Emergency Department (ED) admissions and 7-fold reduction of hospitalizations. After 36 months from Anakinra initiation, 43% of patients had no recurrences, whereas 29% of patients had a single recurrence over the entire period.

During the follow-up, 27% of patients still needed corticosteroid treatment. Regarding adverse events, injection site reactions were common, affecting 38% of patients, but only three patients had to discontinue treatment for this reason; 6% experienced arthralgias and myalgias, 3% showed transient transaminases elevation, 3% had infections, and 1% had transient neutropenia (Table 2); however, these three adverse effects did not require Anakinra discontinuation. As for the effective duration of treatment and tapering protocol, the IRAP study showed that Anakinra treatment longer than 3 months, followed by slow tapering of at least 3 months, was associated with a reduction in the risk of pericarditis recurrence [40].

Overall, AIRTRIP was the first trial to demonstrate a concrete improvement in RP with Anakinra, and the IRAP study assessed its use in a real-world clinical cohort. Although IRAP gave us more information about treatment duration and tapering, these two issues still need to be further explored.

### 4.2. Rilonacept

Rilonacept is a chimeric fusion protein constituted of the ligand-binding domain of human IL-1 receptor (IL-1R1) and the IL-1 receptor accessory protein (IL-1 RAcP) linked to the Fc portion of human IgG1. Circulating rilonacept acts as an “IL-1 trap”, binding both IL-1α and IL-1β, preventing their engagement with the cell surface receptor and downstream inflammatory cascade [41,42]. The Rilonacept half-life is about 7 days, allowing for weekly subcutaneous drug injections [43,44].

Approved for CAPS treatment by the FDA in 2008 [45], Rilonacept recently received FDA approval for treatment of RP after RHAPSODY (Rilonacept Inhibition of Interleukin-1 Alpha and Beta for Recurrent Pericarditis: a Pivotal Symptomatology and Outcomes Study) results from phase II (2020) and phase III (2021) trials [46].

RHAPSODY phase II was a multicenter, open-label study, which enrolled 25 adult patients with idiopathic or post-pericardiotomy RP, symptomatic (at least two pericarditis recurrences) and/or corticosteroid-dependent, with either active or non-active disease. Patients received a 320 mg s.c. Rilonacept loading dose, followed by weekly injections of 160 mg s.c. for 6 weeks; during an optional 18-week treatment-extension period, concomitant anti-inflammatory medications (colchicine, NSAIDs, corticosteroids) were prudently weaned off [46].

Patients with symptomatic RP and elevated CRP had rapid and sustained recovery after Rilonacept treatment, leading to a decrease in pericarditis annual recurrences and improvement in quality of life. Moreover, patients could stop or reduce the dose of at least one concomitant pericarditis medication, without a recurrence. In particular, most patients (84%) receiving corticosteroids at baseline could totally discontinue them, whereas the remaining 16% could reduce them. As for drug safety, 92% of the adverse events were mild–moderate in severity, mostly injection site reactions (Table 2).

The results were confirmed in phase III of the RHAPSODY study, a multicenter, double-blind, placebo-controlled randomized-withdrawal trial enrolling 86 patients (adults or adolescents older than 12 years) with symptomatic RP (with at least two previous recurrences) and elevated CRP (≥1 mg/dL), despite treatment with NSAIDs, colchicine, or steroid therapy.

The majority of patients (85%) had idiopathic RP, whereas post-pericardiotomy pericarditis affected the remainder [46]. During a 12-week run-in period, all patients received Rilonacept 320 mg subcutaneously, followed by a weekly maintenance dose of 160 mg. The run-in period included three periods: a 1-week stabilization period, a 9-week period to wean from pericarditis background therapy, and a 2-week Rilonacept monotherapy period. Among patients receiving Rilonacept monotherapy, 61 had clinical response criteria (CRP ≤ 0.5 mg/dL and weekly NRS score of ≤2) and entered the randomized-withdrawal period to either continue rilonacept or receive a placebo.

In the placebo arm, 23 of 31 (74%) patients experienced recurrence after Rilonacept discontinuation, and all responded to bailout Rilonacept, with no further recurrences; in the Rilonacept arm, 2 of 30 (7%) patients had pericarditis recurrence after temporary interruptions of drug administration.

Injection site reactions and upper respiratory tract infections represented the most common reported adverse events after Rilonacept injections (34% and 23% of participants, respectively), for which only four patients had to discontinue treatment during the run-in period (Table 2).

Overall, compared to the placebo, Rilonacept demonstrated its dramatic efficacy in treatment and prevention of RP in symptomatic patients. As for Anakinra, treatment duration and tapering strategies still represent key questions to explore.

### 4.3. Canakinumab

Canakinumab is a human monoclonal antibody selectively blocking circulating IL-1β, thus forming inactive complexes degraded by the reticuloendothelial system; it has a very long half-life (about 26 days), allowing a drug monthly administration [47]. Currently, Canakinumab is approved by the US FDA and EMA for the treatment of the following inflammatory conditions: CAPS, TRAPS, FMF, hyperimmunoglobulin D syndrome (HIDS)/mevalonate kinase deficiency (MKD), Still’s disease, and gouty arthritis [48,49].

To date, use of Canakinumab in RP is restricted to case reports in the current literature, offering us contrasting results (Table 2).

In 2015, Theodoropoulou et al. reported the use of Canakinumab in a pediatric patient with idiopathic RP, successfully treated with Anakinra for the previous 5 months. When switched to 2 mg/proKg of monthly Canakinumab injections, pericarditis recurred after 1 week and even two times more upon trying higher doses of Canakinumab (4 mg/proKg) and concurrent steroids [50]. Returning to Anakinra treatment led to the patient’s symptoms recovery.

In 2018, Kougkas et al. described three adult patients with RP: two of them with Adult Onset Still Disease (AOSD), the third one with RA. These patients received Canakinumab after the failure of colchicine, methotrexate, corticosteroids, and Anakinra. Two patients with AOSD had remission after initiating Canakinumab 150 mg monthly, thus tapering corticosteroid therapy; however, the RA patient responded only partially to Canakinumab 300 mg monthly, then experienced two relapses [51].

One year later, Epçaçan and colleagues successfully adopted Canakinumab in a child with a clinical history of corticosteroid-dependent RP and anaphylactic reaction to Anakinra [52].

In 2020, Signa et al. reported the unsuccessful use of Canakinumab in two pediatric patients: the first one had RP after pericardiotomy, the second suffered from idiopathic RP. Both of them previously received Anakinra; the first patient discontinued Anakinra after injection site reactions and started Canakinumab 4 mg/kg, later developing relapse. Similarly, the second patient interrupted Anakinra because of poor compliance, switching to 2.5 mg/kg of Canakinumab and relapsing shortly after [53].

Eventually, in 2021, Chawla et al. reported a Canakinumab response in a 31-year-old patient with RP and a past medical history of RA and ulcerative colitis (UC), who had failure with colchicine, prednisone, NSAIDs, Vedolizumab, Adalimumab, and Anakinra [54].

The major effect of Anakinra vs. Canakinumab in treating RP in pediatric patients may lie in the different mechanism of action of these two drugs: Anakinra blocks both IL-1α and IL-1β, whereas Canakinumab exclusively blocks IL-1β [55]. However, this seems no to be true for the patient in the Epçaçan study.

As for the (few) adult case series with RP, the autoimmunity and/or autoinflammatory background seems to influence the response to Canakinumab, with RA representing a negative prognostic risk factor compared to AOSD and CU.

### 4.4. Goflikicept

In 2023, Myachikova and colleagues published early data from an ongoing phase II/III double-blind, randomized-withdrawal, placebo-controlled study adopting a new IL-1 blocker named Goflikicept in patients with RP [56].

In the same way as Rilonacept, Goflikicept is a heterodimer fusion protein binding both IL-1α and IL-1β, with a ten-day half-life. The study by Myachikova et al. allowed enrollment of patients with at least one prior pericarditis recurrence, with either active or inactive pericarditis. Four different periods were analyzed: screening, run-in, randomized withdrawal, and follow-up.

In total, 22 patients participated in the study. The run-in phase found both CRP and chest pain reduction after Goflikicept treatment, consisting of a dosing regimen of 180 mg at day 0, 80 mg at day 7, and an additional dose of 80 mg at day 14. Colchicine and NSAIDs were stopped at day 14 of the run-in phase without tapering, and glucocorticoids were stopped by week 12. Afterwards, 20 patients were equally randomized to either the placebo or Goflikicept group; in the latter, a maintenance dose of 80 mg every two weeks was administered. Recurrence of pericarditis was defined by the presence of two of the following: significant pericardial chest pain, CRP arising, and new or worsening pericardial effusion. No recurrences occurred in the Goflikicept group within 24 weeks after randomization, with respect to 90% recurrences in the placebo group. The safety profile was similar to other IL-1 blockers [56].

While waiting for the final results of Myachikova and colleagues’ study, Goflikicept appears to be an interesting new anti-IL-1 agent in the treatment of RP, although data are very preliminary.

## 5. Interleukin-1 Blockers in Acute Pericarditis

Data regarding the use of IL-1 blockers in AP are scarce, based on poor scientific evidence and only concerning Anakinra (Table 3). There are no available data or ongoing trials for Rilonacept and Canakinumab in this setting. Specifically, in the following evidence, Anakinra was used as the first line or second line, in the absence of colchicine resistance or steroid dependence, differently from what is currently recommended by the guidelines.

In one proof-of-concept trial by Wohlford et al., five patients were enrolled with a diagnosis of AP (according to the ESC criteria) within 24 h of the presentation of chest pain. Three of them presented with their first episode of AP. Four patients were men, and the median age was 38. These patients presented severe pain despite treatment with at least one dose of NSAIDs and colchicine and received a single dose of Anakinra 100 mg within 24 h of presentation. The results after the administration of Anakinra showed a significant reduction in pain at 6 h (*p* = 0.0121) and at 24 h (*p* = 0.0025) compared to the baseline, as measured by pain scales like the Chest Pain Likert scale, and a reduction in the white blood cells count at 24 h (*p* = 0.043)**.**

Limitations of this study included the small sample size and a short observation period after administration. Anakinra was administered in an open-label manner. The study did not include patients with infective pericarditis, malignancy, ischemic pericarditis, or post-procedural pericarditis [57].

Another retrospective study examined 12 adult patients who were admitted to Albany Medical Centre between 2016 and 2018 for AP, whether idiopathic or non-idiopathic. The most common underlying causes of pericarditis in these cases were RA and undifferentiated connective disease. These patients were treated with Anakinra in combination with conventional therapy due to their resistance to or intolerance of conventional treatment. The study excluded patients with malignant or infectious pericarditis or those who had previously been exposed to biologic immunosuppressants. Among the 12 patients treated with both conventional therapy and Anakinra, four had experienced RP, while the remaining patients were dealing with their initial episode of AP. These patients were compared to a control group of 22 individuals who solely received conventional therapy [58].

The results revealed that, in comparison to the control group, all 12 patients in the Anakinra group experienced relief from symptoms upon discharge (*p* = 0.04), and no recurrence of symptoms was observed (*p* = 0.009). During the treatment period, no recurrences were observed in the group receiving both conventional therapy and Anakinra, in contrast to the 38.5% recurrence rate in the control group (*p* = 0.03). The risk of recurrence after discontinuing therapy was similar between the two groups. Additionally, patients receiving Anakinra had a shorter hospital stay (*p* = 0.15). Among the seven patients who were already taking corticosteroids, five successfully tapered off them (71.4%) in the Anakinra group, compared to six out of nine patients (66.7%) in the conventional therapy group (*p* = 0.56).

The study limitations included the retrospective and non-randomized nature, the small sample size, and the absence of a standardized method for assessing symptom improvement [58].

One case report [59] described an 87-year-old man who received a diagnosis of AP caused by a Staphylococcus aureus infection, documented by a positive blood culture and by anti-toxic shock syndrome toxin-1 immunoglobulins E in the patient’s blood, resulting in severe pericardial effusion, as confirmed by echocardiography. Initially, he was treated with antibiotics (oxacillin), ibuprofen, and colchicine, but worsening chest pain and pericardial effusion occurred within five days. Consequently, he was administered Anakinra at a dosage of 100 mg per day, which led to an immediate relief of symptoms and a reduction in pericardial effusion. This approach allowed avoiding pericardiocentesis.

Perna et al. described the case of a 30-year-old patient with COVID-19 vaccine-related AP, as already described in the last two years [7,8]. This patient initially presented at the hospital admission with cardiac tamponade, underwent pericardiocentesis, and then started ibuprofen 600 mg three times a day and colchicine 0.5 mg twice a day, providing symptom relief, allowing discharge. However, he was readmitted to the hospital one week later with fever, chest pain, and an increase in inflammatory markers. At this point, he was treated with Anakinra, starting at 100 mg twice a day on the first day, and subsequently at 100 mg per day. He experienced rapid improvement in symptoms and a reduction in inflammatory biomarkers. After discharge, he continued Anakinra treatment for six months [60].

The role of biological therapy in AP becomes more prominent in cases where adhering to conventional treatments, like NSAIDs and colchicine, becomes considerably challenging, especially in elderly patients. These individuals usually present a series of comorbidities that hinder following the first-line therapy as recommended by the 2015 ESC guidelines.

In this regard, the study conducted by Massaro et al. [61] described the cases of ten patients aged >65 receiving Anakinra, with five experiencing their first episode of AP. Patients had a three-month follow-up and analysis of adverse effects. Two of these patients were 71 and 88 years old, both presenting multiple comorbidities, such as compromised renal function, heart failure, atrial fibrillation, and concurrent use of antiplatelet and anticoagulant therapy. In these cases, conventional therapy with NSAIDs was, therefore, avoided.

Instead, colchicine was introduced at an appropriate dosage, according to renal function and age. Additionally, one patient had psychiatric contraindications for corticosteroid use, while the 71-year-old had shown significant glycemic decompensation following the introduction of such therapy. Considering the worsening of symptoms (including an increase in pericardial effusion with the risk of pericardiocentesis in the 71-year-old patient), the off-label use of Anakinra was then considered.

The result of this intervention was a rapid clinical response without adverse events. The remaining three elderly patients with the first episode of AP experienced worsened pericardial effusion during treatment with NSAIDs in combination with colchicine. Anakinra therapy was then introduced as a rescue therapy to prevent pericardiocentesis. This choice led to a significant reduction in pericardial effusion, thereby avoiding a procedure that in these patients, aged 75, 85, and 87, could be considered even riskier.

The three-month follow-up revealed that one of the patients treated with Anakinra as a rescue therapy died due to heart failure exacerbation. Regarding the risk of recurrence, only one of the remaining patients with AP experienced a relapse, after abruptly discontinuing the biological therapy autonomously. However, restarting Anakinra led to clinical remission once again [61].

## 6. Conclusions

IL-1 plays a key role in both acute and sustained inflammation. RP is a peculiar entity that shares similarities with many other autoinflammatory conditions, for which IL-1 blockers already represent a mainstay of treatment.

In recent years, there has been a growing interest in evaluating the role of these drugs in pericardial disease, particularly when there are suggestive elements of an emerging autoinflammatory pattern.

The available studies show the significant effectiveness of Anakinra in RP, with a good clinical response, an increase in the rate of steroid discontinuation (not without adverse effects when used long term), and a reduction in the rate of further recurrences. The most commonly reported adverse reactions include local reactions at the injection site, arthralgia, myalgia, elevated transaminases, infections, and neutropenia. Regarding the optimal duration of therapy, additional research is required, along with extended long-term follow-up.

Rilonacept shows promising results in terms of clinical response and recurrence reduction. Injection site reactions and upper respiratory tract infections were the most reported adverse events after Rilonacept injections.

The use of Canakinumab in this setting, currently reported only in case studies, presents conflicting results on effectiveness, perhaps due to its selective action only on IL-1β. In fact, Rilonacept and Anakinra act on both IL-1α and IL-1β.

Although both interleukins share the same receptor and pro-inflammatory effect, IL-1α and IL-1β have different biological characteristics. IL-1α represents an “alarmin” that stimulates inflammation, first locally and then systemically. In fact, it is constitutively present in healthy cells. Cellular necrosis causes the release of IL-1α into the extracellular space and binding to its receptor on adjacent cells, with induction of tissue inflammation. In contrast, IL-1β is not constitutively present in healthy cells and is not even biologically active as a precursor, but it is activated after IL-1α -mediated inflammatory stimulus [60]. This could make IL-1α responsible for the vicious cycle of autoinflammation and could explain why drugs that block IL-1β alone do not prevent recurrences.

Moreover, as mentioned above [18,19,20], the two different pathways of RP (autoimmune vs. autoinflammatory), determine two different disease phenotypes, usually requiring different therapeutic approaches [20]. Indeed, adult patients’ rheumatological background might influence the response to Canakinumab in RP.

Our study has potential limitations. First, an extensive literature review was performed, but not a meta-analysis. Furthermore, especially regarding AP, we have included small observational studies or case reports due to the limited data currently available. This limitation causes a difficult final interpretation of the use of IL-1 blockers in the acute phase.

More studies are needed to evaluate the efficacy of IL-1 blockers in AP, with few case reports existing showing interesting results.

Although current European guidelines for the use of these drugs in pericardial disease were published in 2015, the past 8 years have seen numerous studies highlighting the effectiveness and safety of this therapy, to the extent of suggesting a more significant role of anti-IL-1 drugs in upcoming guidelines.

Moreover, conventional treatment is not devoid of side effects; for specific patient groups, like the elderly, managing multiple comorbidities poses a challenge, thus emphasizing the pressing need for new therapeutic alternatives. This approach is not just effective in recurrent cases, but also during a first episode of AP, where conventional therapy is contraindicated or ineffective. In this scenario, it could aid in avoiding invasive procedures, such as pericardiocentesis, by directly addressing a key element of pericardial inflammation.

## Figures and Tables

**Table 1:** Therapeutic options for recurrent pericarditis by ESC 2015 guidelines.

Drugs	Mechanism	Indications	Note
Aspirin	COX inhibition	First line	As an adjunct to colchicine
Ibuprofene	COX inhibition	First line	As an adjunct to colchicine
Indomethacin	COX inhibition	First line	As an adjunct to colchicine
Colchicine	Inhibition of inflammasome and neutrophil functions	First line	As an adjunct to NSAIDs
Corticosteroids	Antinflammatory and Immunosuppressive action	Second line	If NSAIS/colchicine are contraindicated and after exclusion of infectious causes
Anakinra	IL-1 receptor antagonist	Third line	In cases of corticosteroid-dependent recurrent pericarditis in patients not responsive to colchicine
Azathioprine	Purine synthesis inhibitor	Third line	In cases of corticosteroid-dependent recurrent pericarditis in patients not responsive to colchicine
Immunoglobulins	Immunomodulatory and anti-infective agents	Third line	In cases of corticosteroid-dependent recurrent pericarditis in patients not responsive to colchicine

COX: Cyclooxygenase.

**Table 2:** Summary of studies on the use of anti-IL-1 drugs in recurrent pericarditis.

Drug	Study	Design	Year	Pts n.	Dose	Results	AE
Anakinra	AIRTRIP	PT	2016	21	2 mg/kg/day	↓ recurrence	ISR; ↑ALT; ↑AST
	IRAP	PT	2020	224	100 mg/day	↓ recurrence	ISR; A; M
Rilonacept	RHAPSODY II	PT	2020	25	320 mg LD and then 160 mg/week	↓ recurrence	ISR; I
	RHAPSODY III	PT	2021	86	320 mg LD 160 mg/week	↓ recurrence	ISR; URTI
Canakinumab	Theodoropoulou	CR	2015	1	2 mg/kg/month and then 4 mg/kg/month	Relapse	Unreported
	Kougkas	CR	2018	3	150/mg/month	Remission (2 Pts) Relapse (1 Pts)	Unreported
	Epçaçan	CR	2019	1	5 mg/kg/month	Remission	None
	Signa	CR	2020	2	2.5 mg/kg/month or 4 mg/kg/month	Relapse	Unreported
	Chawla	CR	2021	1	Unknown	Remission	Unreported

Pts: Patients; n: number; AE: Adverse Effects; PT: Prospective Trial; LD: Loading Dose; CR: Case Report; ISR: Injection site reaction; A: Arthralgias; M: Myalgias; I: Infections; URTI: Upper Respiratory Tract Infection; ALT: Aminotransferase Alanine Transaminase; AST: Aspartate Transaminase; ↓: decrease; ↑: increase.

**Table 3:** Summary of studies on the use of anti-IL-1 drugs in acute pericarditis.

Drug	Study	Design	Year	Pts n.	Dose	Results	AE
Anakinra	Wohlford	COLT	2020	5	100 mg single dose	↓ pain ↓ WBC	None
	Shaukat	RS	2020	8	100 mg no tapering	↓ symptoms ↓ HS	None
	Sicignano	CR	2021	1	100 mg for 5 days	↓ effusion Pericardiocentesis avoided	None
	Perna	CR	2022	1	100 mg/day with tapering	↓ symptoms ↓ inflammation	None
	Massaro	CS	2023	5	100 mg/day with tapering	Stable remission	None

Pts: Patients; n: number; AE: Adverse Effects; COLT: Clinical Open Label Trial; RS: Retrospective Study; HS: Hospital Stay; CR: Case Report; CS: Case Series; ↓, decrease.

## Data Availability

No new data were created or analyzed in this study. Data sharing is not applicable to this article.
